# Horizon-Aware Forecasting of Hypertension Prevalence and Mean Total Cholesterol in Chinese Men: Retrospective Leakage-Controlled Benchmark Study Comparing Statistical, Machine-Learning, and Neural Models

**DOI:** 10.2196/99686

**Published:** 2026-11-11

**Authors:** Weiguo Lai, Fazli Subhan, Nana Zhou, Jawad Khan

**Affiliations:** 1 School of Computer Sciences, Shandong Xiehe University, Jinan, Shandong, China; 2 School of Information and Artificial Intelligence, Yangzhou University, Yangzijin Campus, No. 196 Huayang West Road, Yangzhou, Jiangsu, 225127, China, 86 17851975652

**Keywords:** hypertension, cholesterol, annual time series, expanding-window evaluation, leakage control, machine learning, neural forecasting, reproducibility, model comparison

## Abstract

**Background:**

Forecasts of population-level cardiovascular risk factors can support surveillance and planning, but annual country-level health series are often short. With only 30 or 39 annual values per target, model flexibility can outpace the information available for training, making leakage control, repeated temporal evaluation, and comparison with simple baselines essential.

**Objective:**

The aim of this study is to compare statistical, classical machine-learning, and neural forecasting models for age-standardized prevalence of hypertension (ASPH) and mean total cholesterol (MTC) in Chinese men and to test whether attention or substantially greater neural capacity provides consistent predictive benefit.

**Methods:**

The authors analyzed 2 officially sourced annual series: ASPH for Chinese men aged 30 to 79 years (1990‐2019; 30 observations) and MTC for Chinese men aged 18 years or older (1980‐2018; 39 observations). No series was interpolated, extrapolated, imputed, smoothed, spliced, or synthetically extended. Leakage-controlled expanding-window evaluation began after 21 observations and assessed 1-, 2-, and 5-year horizons. Classical machine-learning and neural models used a fixed primary lookback of 3 (L=3), with nested lookback selection disabled; neural models used 10 fixed seeds. Performance was assessed with root mean square error (RMSE), mean absolute error, mean absolute scaled error, root mean squared scaled error, symmetric mean absolute percentage error, directional accuracy, mean error, median error, diagnostic *R*², prespecified neural-ablation inference, empirical forecast-interval coverage, and computational-cost reporting.

**Results:**

Statistical models had the lowest RMSE in all 6 target-horizon cells. Autoregressive integrated moving average won for ASPH at h=1 (RMSE 0.000178), h=2 (RMSE 0.000578), and h=5 (RMSE 0.004324), and for MTC at h=1 (RMSE 0.000400) and h=2 (RMSE 0.001479); error-trend-seasonal state space won for MTC at h=5 (0.006991). Classical machine-learning and primary neural models won no cells. Removing attention reduced RMSE in 5 of 6 ablation cells, while the compact architecture had lower RMSE than Original23K in 5 of 6 cells; formal differences were detected for both contrasts at ASPH h=1 and h=2, whereas ASPH h=5 (n=5) was descriptive only. Predictive intervals often undercovered. The benchmark completed all 7242 planned output-producing fits.

**Conclusions:**

Simple statistical forecasting remained highly competitive in these short, retrospectively estimated annual series. Greater complexity did not ensure better point prediction, and neither attention nor larger capacity conferred a consistent advantage. Point accuracy did not ensure well-calibrated predictive uncertainty; findings were conditional on the 2 targets, target-specific horizons, and small evaluation samples.

## Introduction

### Background and Rationale

Population-level trajectories of hypertension and cholesterol remain central to cardiovascular surveillance, prevention planning, and long-range health policy [1-3]. Forecasting these indicators is nevertheless difficult when the available records are annual and short. The official series used here contain 30 age-standardized prevalence of hypertension (ASPH) values and 39 mean total cholesterol (MTC) values, providing few independent temporal examples, and under these conditions, a flexible model can fit historical windows well without delivering stable out-of-sample gains [4,5].

The broader forecasting literature also cautions against assuming that model complexity translates directly into predictive superiority. Large forecasting competitions and public benchmark archives show that simple statistical baselines, carefully designed combinations, and parsimonious models can remain highly competitive across heterogeneous datasets [6-9]. This evidence does not imply that simple models always win; rather, it makes benchmark design itself part of the scientific question. Fair comparison requires common forecast origins, horizon-specific tasks, leakage-free preprocessing, and evaluation against credible low-variance baselines.

### Related Forecasting Literature and Methodological Gap

Reliable time-series evaluation must preserve temporal order and keep configuration choices separate from final performance estimation. Rolling-origin and blocked temporal validation have long been recommended for forecasting [10-12], while research on selection bias shows that repeated tuning against an evaluation sample can produce optimistically biased estimates, especially when data are scarce [13]. Future observations can also differ from historical training data, a concern studied in out-of-distribution time-series forecasting [14]. For short annual health series, these issues make a prespecified temporal protocol, fold-local preprocessing, and cautious uncertainty reporting particularly important.

Modern forecasting research spans recurrent probabilistic models, basis-expansion networks, multi-horizon attention architectures, transformers, large-language-model adaptations, and time-series foundation models [5,15-23]. These approaches demonstrate the benefits of representation learning and large-scale pretraining, but most are developed using long sequences, many related series, or very large pretraining corpora. Recent work on lightweight architectures further shows that strong forecasting performance need not require large parameter counts [24]. Consequently, results from large-scale benchmarks cannot be assumed to transfer directly to annual target series containing only 30 or 39 values.

Health care forecasting studies likewise range from longitudinal electronic health records and intensive-care time series to recent evaluations of time-series foundation models for vital-sign forecasting [25-27]. Those settings are clinically important but differ materially from population-level annual surveillance, where the core challenges are extreme sample scarcity, long forecast horizons, and extrapolation. A specific gap, therefore, remains: few studies compare statistical, classical machine-learning (ML), and neural approaches under one leakage-controlled, horizon-aware protocol for fully sourced, short, annual population-health indicators while also separating point accuracy, architectural ablation, initialization variability, and predictive-interval calibration.

### Study Objective and Contribution

Accordingly, the contribution of this study is not a new neural architecture but rather a rigorous benchmark for short annual health series. We ask how forecasting performance changes across 2 cardiovascular risk indicators and 3 forecast horizons while enforcing temporal leakage controls and keeping numerical ranking, prespecified ablation inference, initialization variability, and predictive uncertainty conceptually distinct.

- A leakage-controlled expanding-window benchmark across statistical, classical ML, and neural families, using official source coverage and a prespecified evaluation protocol.
- A horizon-specific evaluation at h=1, h=2, and h=5, with a common 21-observation initial history and a fixed lookback of L=3 for ML and neural models.
- A reproducibility and model-complexity analysis using 10 neural seeds, prespecified attention and capacity ablations, valid paired statistical comparisons, empirical interval coverage across all 6 cells, archived predictions, and reconciled computational-cost reporting.

Accordingly, this study aimed to determine how statistical, classical ML, and neural models compare across 2 officially sourced cardiovascular risk indicators and 3 forecast horizons under a common leakage-controlled design.

Figure 1 provides an integrated overview of the official data foundation, leakage controls, preprocessing routes, model families, shared evaluation, principal findings, and reproducibility components of the benchmark.

**Figure 1.**
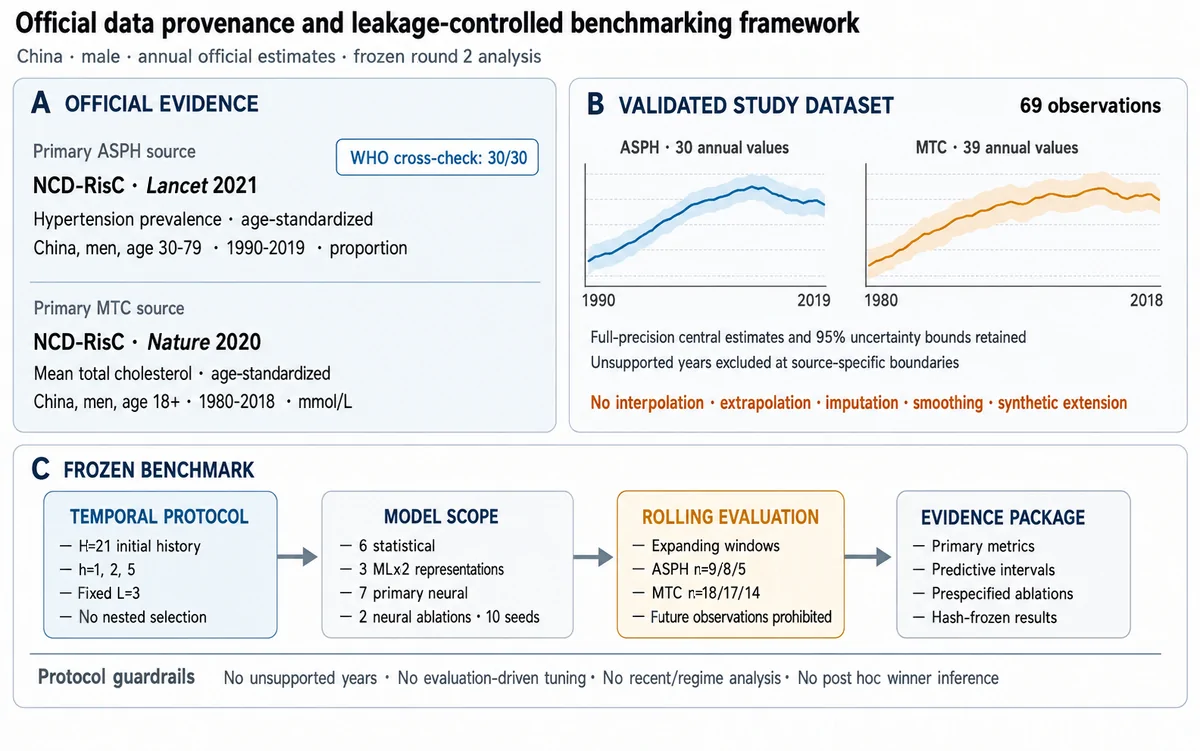
Official data provenance and leakage-controlled benchmarking framework. (A) Primary NCD Risk Factor Collaboration (NCD-RisC) hypertension and cholesterol data sources, with the World Health Organization (WHO) hypertension indicator used as a transcription and rounding cross-check. The analysis used official China-male annual estimates only. Age-standardized prevalence of hypertension (ASPH) was reconstructed from the full-precision NCD-RisC *Lancet* 2021 [2] country dataset for adults aged 30 to 79 years (1990‐2019); the WHO Global Health Observatory (GHO) 608DE39 indicator was retained only as a transcription and rounding consistency check of the NCD-RisC–derived ASPH values, not as an independent source. Mean total cholesterol (MTC) was reconstructed from the NCD-RisC *Nature* 2020 [3] country dataset for adults aged 18 years and older (1980‐2018). (B) Reconstructed ASPH series (30 annual observations, 1990-2019) and MTC series (39 annual observations, 1980-2018), with source-specific uncertainty bounds retained. Unsupported years were excluded, and no interpolation, extrapolation, imputation, smoothing, splicing, or synthetic extension was performed. The validated 69-observation dataset was evaluated under the frozen protocol using statistical, classical machine-learning, primary neural, and prespecified neural-ablation models. (C) The prespecified temporal protocol, model scope, rolling-origin evaluation, leakage controls, and reproducibility evidence package. ML: machine learning.

## Methods

### Study Design

This retrospective benchmarking study evaluated forecasting methods using the same annual population-level series, forecast origins, target years, and horizons. All reported results were derived from the finalized, prespecified evaluation outputs. The analysis was predictive rather than causal: it did not estimate treatment effects, individual risk, or intervention impact.

### Data Sources and Provenance

For ASPH, we used the full-precision country estimates released by NCD Risk Factor Collaboration (NCD-RisC) in its 2021 *Lancet* hypertension study [2]. We selected China, men, ASPH among adults aged 30 to 79 years, and all 30 annual central estimates from 1990 through 2019. Lower and upper 95% uncertainty bounds were retained, and source percentages were divided by 100 for analysis in proportion units. The World Health Organization (WHO) Global Health Observatory (GHO) hypertension indicator (608DE39) was used only for a transcription and rounding consistency check against the NCD-RisC–derived ASPH values, not as an independent source or as the primary ASPH source.

For MTC, we used the NCD-RisC country estimates released with the 2020 *Nature* cholesterol study [3]. We selected China, men, adults aged 18 years or older, and all 39 annual MTC estimates from 1980 through 2018. Central estimates and lower and upper 95% uncertainty bounds were retained without transformation in mmol/L; high-density lipoprotein and non–high-density lipoprotein cholesterol were not used as the target.

### Provenance Verification and Series Reconstruction

After equivalent rounding to 1 decimal percentage point, the WHO GHO hypertension indicator (608DE39) agreed with the NCD-RisC ASPH series for all 30 years (1990‐2019). WHO’s metadata attribute the indicator’s estimates to the same NCD-RisC 2021 pooled hypertension analysis used for ASPH, so this agreement confirms transcription and rounding consistency rather than independent corroboration. This in-period consistency check provides no evidence for years outside the official NCD-RisC source period (1990‐2019).

Unsupported observations were excluded: ASPH values for 1970 through 1989 and 2020 through 2023, and MTC values for 1970 through 1979 and 2019 through 2023. We did not interpolate, extrapolate, impute, smooth, splice, or synthetically extend either series. The reconstructed study dataset therefore contained 69 observations: 30 for ASPH and 39 for MTC.

The NCD-RisC annual series comprise retrospective, model-based population estimates generated from pooled population-representative evidence. They are not raw, independently observed annual surveys. This benchmark forecasts the published series of annual estimates rather than direct annual survey measurements.

The forecasting pipeline was controlled for temporal leakage at every evaluation origin; however, the source observations are retrospective estimates rather than historical real-time data vintages. This distinction limits its interpretation as a simulation of forecasts made from information releases available in real time.

### Temporal Evaluation Design

Evaluation began after 21 annual observations were available for each target (initial history H=21). At each forecast origin, the training window contained only observations available up to and including that origin and then expanded by one observation [10,28]. Direct forecasts were evaluated separately at horizons h=1, h=2, and h=5.

For ASPH (1990‐2019), the first origin was 2010. The h=1 targets were 2011 through 2019 (n=9), the h=2 targets were 2012 through 2019 (n=8), and the h=5 targets were 2015 through 2019 (n=5).

For MTC (1980‐2018), the first origin was 2000. The h=1 targets were 2001 through 2018 (n=18), the h=2 targets were 2002 through 2018 (n=17), and the h=5 targets were 2005 through 2018 (n=14). Figure 2 summarizes the frozen design.

**Figure 2.**
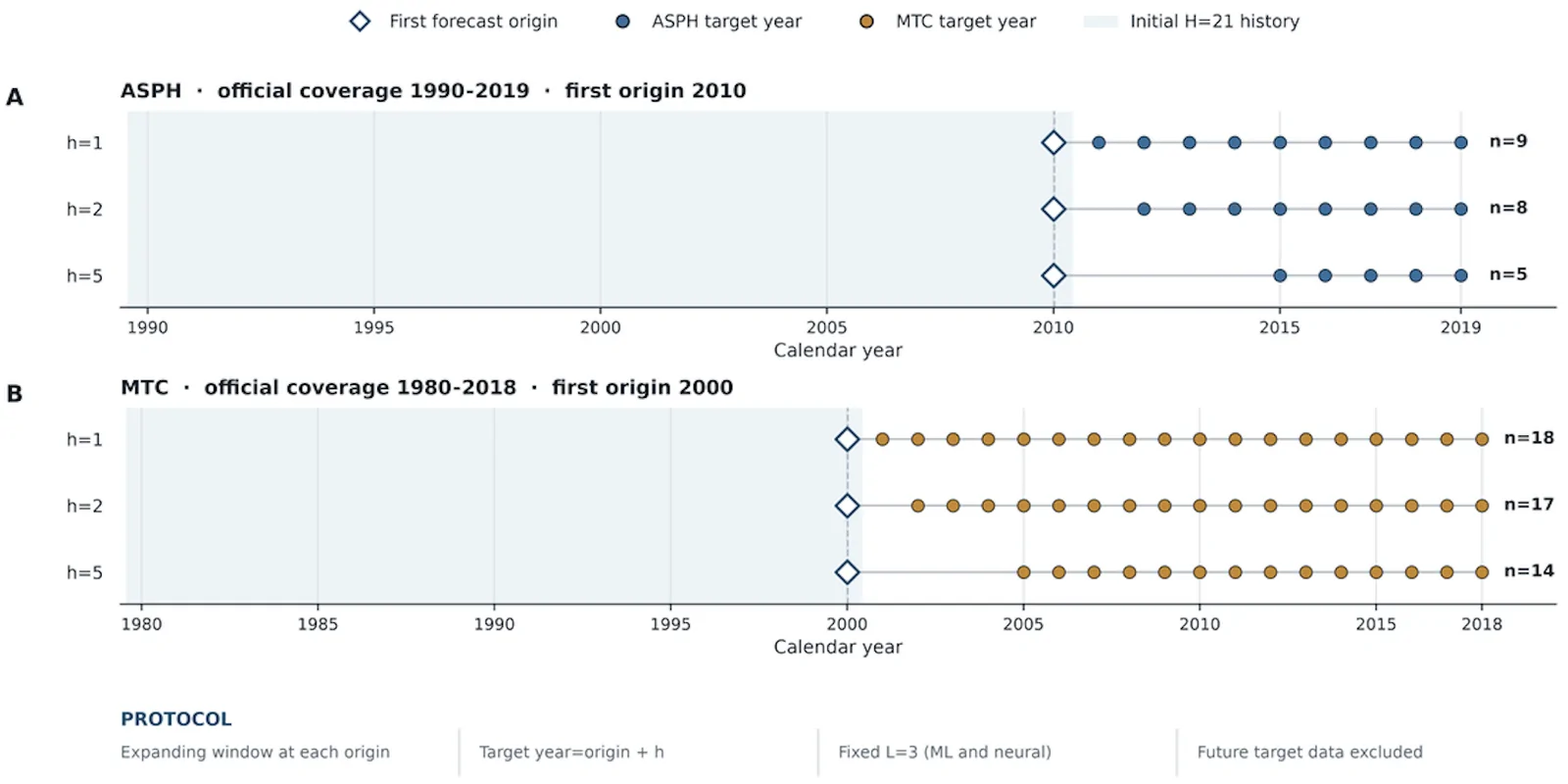
Frozen, leakage-controlled, rolling-origin evaluation design. (A) Age-standardized prevalence of hypertension (ASPH) rolling-origin evaluation for hypertension prevalence, with official coverage from 1990 to 2019, first forecast origin in 2010, and forecasting horizons of 1, 2, and 5 years (n=9, 8, and 5, respectively). (B) Mean total cholesterol (MTC) rolling-origin evaluation for mean total cholesterol, with official coverage from 1980 to 2018, first forecast origin in 2000, and the same forecasting horizons (n=18, 17, and 14, respectively). Both targets used an expanding-window evaluation beginning after 21 observed annual values. The protocol information below the panels A and B describes the expanding-window evaluation, target-year calculation, fixed lookback, and exclusion of future target data. Classical machine-learning (ML) and neural direct-horizon models used a fixed lookback of L=3 before evaluation; nested lookback selection was disabled. No future observations entered fitting or preprocessing, and no recent-period, regime-aware, or unsupported-year analyses were conducted.

### Forecast Construction and Leakage Controls

For a forecast origin t and horizon h, the direct target was the value at t+h. The feature window was formed only from observations through t, and the target was never included in the feature window. A first-difference representation predicted the change relative to the last observed value and reconstructed the level by adding that change to the origin value. A linear-detrended representation fit a time trend within the training fold, modeled the residual component, and added the fold-fitted trend back after prediction. These operations were repeated independently at every origin.

All scaling and transformation parameters were estimated using only the training data available at the current origin. No global scaler, future maximum or minimum, future trend, or future residual information was available to any model [11-13]. The ML and neural lookbacks were fixed at L=3 before evaluation. Nested lookback selection was disabled, and alternative lookbacks were not part of the finalized benchmark.

The statistical baselines used the same origin and horizon definitions as those in ML and neural models. This makes a 1-year naive forecast, a 5-year autoregressive integrated moving average (ARIMA) forecast, and a neural direct forecast comparable at the level of the observed target year, while avoiding the common error of treating a multi-horizon forecast path as if it were 6 independent one-step forecasts. Forecast records retain the origin year, target year, horizon, model family, representation, and seed, where applicable, allowing every reported aggregate to be recomputed from the common rows.

### Statistical Models

The statistical candidates were naive, drift, linear trend, error-trend-seasonal state space (ETS), ARIMA, and theta, representing commonly used low-variance benchmarks in empirical forecasting studies [6-8,28]. Naive repeats the last observed value; drift extrapolates the average historical change; linear trend fits a least-squares temporal trend; and ETS, ARIMA, and theta model complementary forms of level, trend, and serial structure. Every model was refit using only the training data available at each origin, with selection performed separately for each target and horizon.

### Classical ML Development

The classical ML candidates were support vector regression (SVR), random forest, and extreme gradient boosting (XGBoost) [29-31]. Two direct-horizon representations were evaluated with a fixed lookback of L=3: first difference, which predicts a horizon-specific change and reconstructs the level from the last available observation, and fold-local linear detrending, which predicts the residual component and restores the fold-fitted trend.

Representations, scalers, and model settings were applied using the training data available at each origin. Direct horizon-specific targets kept the h=1, h=2, and h=5 tasks separate.

No configuration was selected or reselected based on evaluation-target performance. Consequently, family-level comparisons remained descriptive and were not converted into an evaluation-period hyperparameter search.

The primary lookback (L=3) and model settings were fixed before evaluation. Alternative lookbacks were not evaluated, and no nested or evaluation-driven lookback selector was used.

### Neural-Model Development and 10-Seed Evaluation

The 7 primary neural architectures were long short-term memory (LSTM), gated recurrent unit (GRU), bidirectional LSTM (BiLSTM), causal convolutional neural network (CNN), CNN-LSTM, CNN-GRU, and a CNN-BiLSTM compact attention model. Together, they represent recurrent, bidirectional, convolutional, hybrid, and attention-based sequence models [5,32-36]. The goal was not to claim exhaustive architectural coverage but to compare several commonly used design families under the same temporal protocol.

Two additional architectures were used only for diagnostic ablations: a compact CNN-BiLSTM model without attention and the original 22,369-parameter attention architecture. The compact attention model contained 857 trainable parameters, and the no-attention comparator contained 569. The larger model was retained to test the effect of capacity rather than as headline architecture. Parameter counts, dropout, lookback, representation, and epoch budgets were taken from the finalized configuration records.

Neural architectures, representations, optimizer settings, dropout, and epoch budgets were frozen before evaluation. Outer fits used all eligible historical supervised samples available at the current origin, with neither a final validation split nor early stopping. Evaluation target years were not used to select configurations or epochs [13].

All primary and ablation neural direct-horizon fits used the fixed L=3 lookback. Nested and evaluation-driven lookback selections were disabled. Full configuration details are documented in the frozen public release.

Each architecture-target-horizon cell was evaluated with 10 prespecified seeds (3101‐3110). Seed spread measures the sensitivity to random initialization around a fixed configuration; it is not predictive uncertainty and was not interpreted as a calibrated forecast interval.

For primary neural performance, predictions were averaged target-by-target across the 10 fixed seeds before RMSE and other forecasting metrics were computed; these ensemble-mean prediction metrics defined the neural values used in the primary cross-model ranking.

### Performance Metrics

RMSE was used to emphasize larger forecast errors, mean absolute error (MAE) to summarize absolute error in the target’s native units, and mean absolute scaled error (MASE) to scale absolute error against the corresponding in-sample one-step naive benchmark [37]. Root mean squared scaled error (RMSSE) provided the analogous squared-error scaling, while symmetric mean absolute percentage error, mean error, median error, and directional accuracy were retained as complementary diagnostics. Because no single metric captures every aspect of forecast quality, model rankings were interpreted jointly rather than relying on one score alone.

MASE and RMSSE are scale-free forecast-error measures benchmarked against in-sample naive errors [37]. *R*² was included only as a diagnostic measure of out-of-sample performance relative to a constant-mean-variance reference over each evaluation window; it is not an in-sample model-fit metric and was not used for primary ranking.

Directional accuracy, mean error, and median error were retained as secondary diagnostics for the interpretation of forecast direction and bias. The headline ranking remained based on the RMSE, MAE, and MASE.

### Statistical Comparisons

Forecast errors were paired by common origin. Formal paired comparisons were limited to 2 prespecified neural ablation families: compact attention vs no attention, and compact attention vs Original23K. Where mathematically valid, the Diebold-Mariano test used the Harvey-Leybourne-Newbold (HLN) finite-sample modification and horizon-minus-one autocovariance truncation, with Holm adjustment applied separately within each ablation family [38-40]. Reported paired-bootstrap intervals for inferentially valid cells used 5000 repetitions; no inferential interval is reported for ASPH at h=5 because formal inference was disabled a priori.

The reporting vocabulary distinguishes a numerical difference from an inferential result. A result is described as a statistically detected difference only when the corrected comparison supports this conclusion. Otherwise, it is described as no detected difference with limited power. A nonsignificant result is not converted into an equivalence claim.

### Uncertainty Analysis

Predictive uncertainty and neural initialization variability were analyzed separately. Predictive intervals were available for the 6 statistical baselines and for classical ML specifications when sufficient calibration history was available; no neural predictive-interval method was frozen. Linear trend used ordinary least squares observation-prediction intervals from Statsmodels (Python Software Foundation), and ARIMA used its native forecast intervals. Theta used native Statsmodels prediction intervals when finite, with training first-difference quantile intervals as a fallback. Naive intervals used centered empirical quantiles of training-only, horizon-specific naive forecast errors; drift used centered empirical quantiles of horizon-specific errors generated from prior expanding prefixes; ETS used centered quantiles of its selected fit’s training residuals. These empirical statistical intervals required at least 8 finite residuals. For each classical-ML direct-horizon forecast, a symmetric rolling finite-sample conformal interval was centered on the point forecast and calibrated using absolute, horizon-specific out-of-fold errors from up to the 8 most recent pseudo-origins with at least 20 training observations and target years no later than the outer forecast origin. At least 8 finite calibration errors were required, so early intervals could be unavailable. Empirical coverage was evaluated at the nominal 80% and 95% levels [41]. Coverage was summarized as both the unweighted mean of specification-level coverage and the pooled proportion across valid model-forecast intervals. The analysis also reports each primary winner’s mean interval width at both levels, calculated as the upper minus the lower across valid forecasts. Neural seed variability was not treated as a predictive interval.

### Computational Environment and Reproducibility

Computations were performed without a graphics processing unit on an Intel Core Ultra 9 185H system with 16 physical and 22 logical cores and approximately 33.7 GB of RAM. Neural cells used 18 worker processes with one computational thread per process. Timing records distinguished overall wall-clock duration, cumulative per-fit elapsed time, and cumulative estimator-fitting time; the reproducibility manifests retained fit identifiers, configurations, and failure records.

### Ethical Considerations

This forecasting study was a secondary analysis of officially sourced aggregate annual population-level estimates. No separate institutional ethics-review application was submitted for this analysis because it involved no direct participant interaction or intervention and used no individual-level records or identifiable personal information. Article 32(2) of the 2023 Chinese national Measures for Ethical Review of Life Science and Medical Research Involving Humans lists research using anonymized data among the categories that may be exempt from ethics review [42]. This policy-based rationale is not presented as a formal institutional exemption, and no ethics approval or exemption identifier applies to this analysis.

No additional informed consent was obtained for this analysis because the authors did not recruit participants or access individual-level or identifiable participant data. This statement concerns only the present aggregate secondary analysis; it does not assert that the current authors obtained original participant consent, that an ethics committee formally waived consent, or that the complete compiled series has a single original ethics approval. The analytical dataset contained no individual-level records, direct identifiers, or identifiable personal information; therefore, no participant identity was accessible during analysis. No participants were recruited or compensated for this study. All manuscript and supplementary figures are analytical displays and contain no identifiable individuals; therefore, image consent is not applicable.

## Results

### Analysis Set and Evaluation Counts

The reconstructed analysis contained 69 official annual estimates: 30 ASPH values for 1990 through 2019 and 39 MTC values for 1980 through 2018. For ASPH, h=1, h=2, and h=5 consisted of 9, 8, and 5 forecast targets; for MTC, the corresponding counts were 18, 17, and 14. The five ASPH h=5 forecasts provide an especially small and unstable basis for comparison and are reported descriptively.

### Statistical Baseline Performance

Statistical models achieved the lowest RMSE in all 6 target-horizon cells. ARIMA won for ASPH at h=1, h=2, and h=5, and MTC at h=1 and h=2; ETS won for MTC at h=5. These are cell-specific results from 2 short annual series and do not establish universal statistical-model superiority.

### Stationarity-Aware ML Performance

Among classical ML specifications, the lowest-RMSE models were SVR for ASPH h=1, XGBoost for ASPH h=2, random forest for ASPH h=5, SVR for MTC h=1 and h=2, and random forest for MTC h=5. No model was the overall winner in its target-horizon cell. The comparison retained both first-difference-direct and fold-local detrend-direct specifications at fixed L=3.

The ML results illustrate why representation is part of the scientific comparison. In the frozen descriptive ranking, first-difference classical-ML specifications ranked 3rd to 12th of 19 and had lower RMSE than their same-model detrended counterparts in all 18 target-horizon comparisons; detrended classical-ML specifications ranked 12th to 18th. The primary ranking therefore compared fixed, leakage-controlled specifications rather than choosing a transformation after observing evaluation performance.

### Ten-Seed Neural Performance and Architecture Ablations

The lowest-RMSE primary neural specifications were BiLSTM for ASPH h=1, causal CNN for ASPH h=2, CNN-GRU for ASPH h=5, causal CNN for MTC h=1 and h=2, and CNN-LSTM for MTC h=5. No primary neural specification won an overall cell. The 10-seed distributions in Figure 3 describe initialization sensitivity only. In prespecified ablations, the no-attention configuration had lower RMSE in 5 of 6 cells, and the compact architecture had lower RMSE than Original23K in 5 of 6 cells.

**Figure 3.**
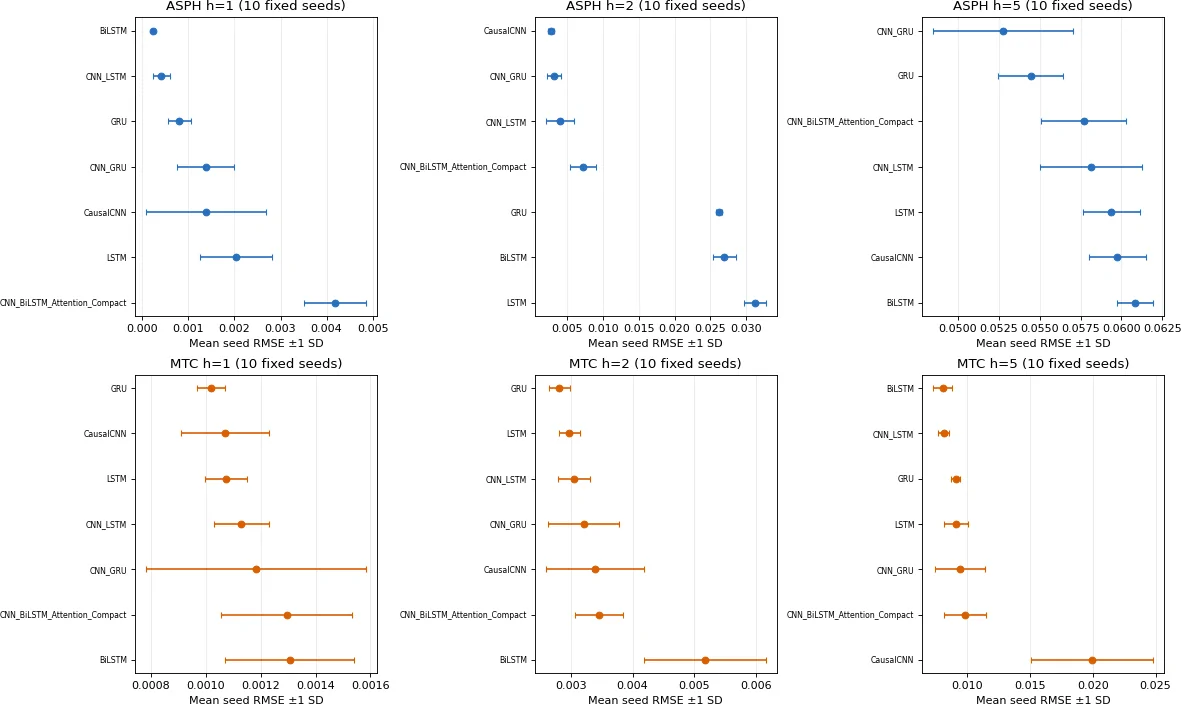
Neural initialization variability across targets and forecast horizons for the 7 primary neural architectures. For each architecture and target-horizon cell, root mean square error (RMSE) was calculated separately for each of the 10 fixed seeds across that cell’s forecast targets. Points show the arithmetic mean of those 10 seed-specific RMSE values; horizontal bars show ±1 sample SD across these values. This mean-per-seed-RMSE summary is distinct from the primary neural performance metrics, which were calculated after averaging predictions, target-by-target, across the 10 fixed seeds. The top rows show age-standardized prevalence of hypertension (ASPH) at h=1, h=2, and h=5, and the bottom rows show mean total cholesterol (MTC) at the same horizons. Seed dispersion describes sensitivity to random initialization under a fixed architecture and training protocol; it is not predictive uncertainty. BiLSTM: bidirectional long short-term memory; CNN: convolutional neural network; GRU: gated recurrent unit; LSTM: long short-term memory.

### Final Cross-Family Primary Comparison

Table 1 reports the lowest-RMSE model within each family and the overall winner for every target-horizon cell. Figure 4 plots actual RMSE for all 19 primary specifications across the 6 target-horizon panels.

**Table 1.** Primary cross-family results by target and horizon.^a^

Target	Horizon	Best statistical	Best ML^b^	Best neural	Overall winner	RMSE^c^	MAE^d^	MASE^e^
ASPH^f^	1	ARIMA^g^	SVR^h^—first difference	BiLSTM^i^	ARIMA	0.000178	0.000155	0.031
ASPH	2	ARIMA	XGBoost^j^—first difference	Causal CNN^k^	ARIMA	0.000578	0.000512	0.101
ASPH	5	ARIMA	Random forest—first difference	CNN-GRU^l^	ARIMA	0.004324	0.003877	0.754
MTC^m^	1	ARIMA	SVR—first difference	Causal CNN	ARIMA	0.000400	0.000305	0.024
MTC	2	ARIMA	SVR—first difference	Causal CNN	ARIMA	0.001479	0.001166	0.090
MTC	5	ETS^n^	Random forest—first difference	CNN-LSTM^o^	ETS	0.006991	0.006203	0.473

a Candidate-family columns identify the lowest-RMSE specification in each family. RMSE, MAE, and MASE are reported for the overall winner. ASPH is analyzed as a proportion, and MTC is measured in millimoles per liter. Rankings are based on ascending RMSE, then MAE, and then MASE.

b ML: machine learning.

c RMSE: root mean square error.

d MAE: mean absolute error.

e MASE: mean absolute scaled error.

f ASPH: age-standardized prevalence of hypertension.

g ARIMA: autoregressive integrated moving average.

h SVR: support vector regression.

i BiLSTM: bidirectional long short-term memory.

j XGBoost: extreme gradient boosting.

k CNN: convolutional neural network.

l GRU: gated recurrent unit.

m MTC: mean total cholesterol.

n ETS: error-trend-seasonal state space.

o LSTM: long short-term memory.

**Figure 4.**
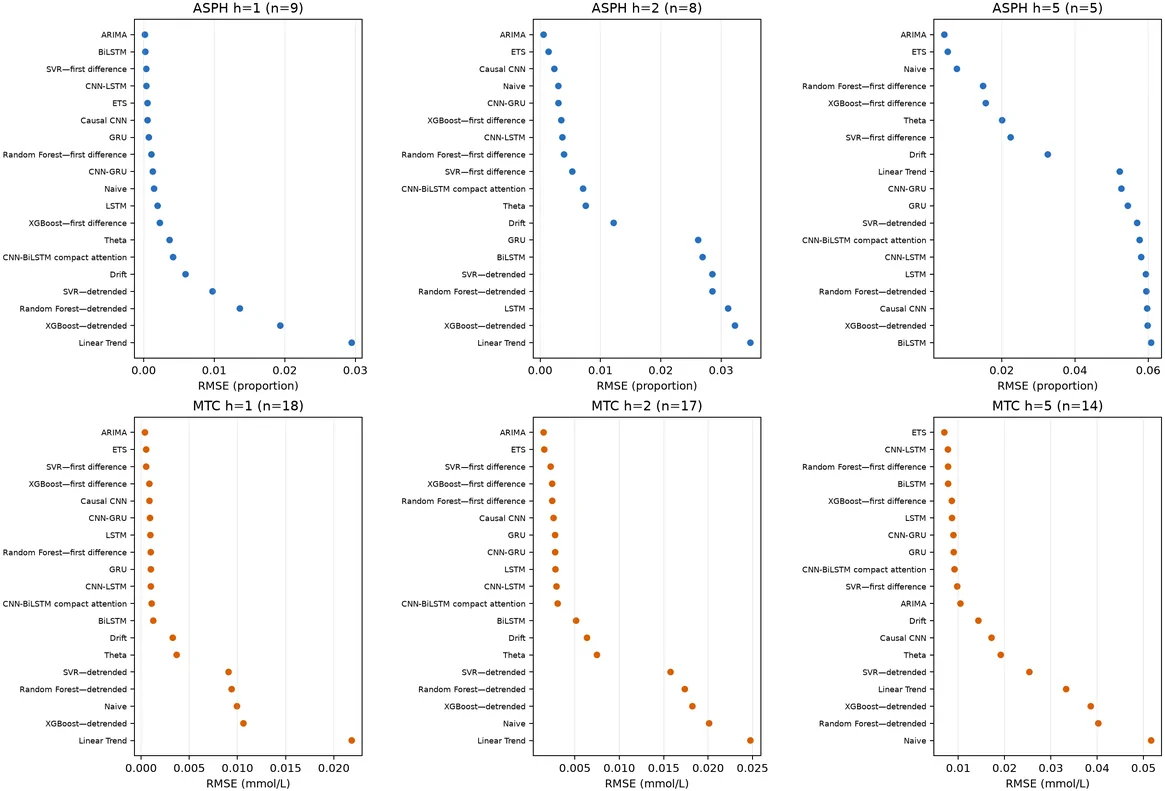
Primary root mean square error (RMSE) comparison across all model specifications, targets, and forecast horizons. The 6 panels plot actual RMSE for all 19 primary specifications for age-standardized prevalence of hypertension (ASPH) at h=1 (n=9), h=2 (n=8), and h=5 (n=5), and for mean total cholesterol (MTC) at h=1 (n=18), h=2 (n=17), and h=5 (n=14). ASPH RMSE is shown in proportion units, and MTC RMSE is expressed in millimoles per liter; lower RMSE is better. Autoregressive integrated moving average (ARIMA) had the lowest RMSE in 5 cells, and error-trend-seasonal state space (ETS) had the lowest RMSE for MTC h=5. ASPH h=5 contains only 5 forecasts and is extremely unstable. BiLSTM: bidirectional long short-term memory; CNN: convolutional neural network; GRU: gated recurrent unit; LSTM: long short-term memory; SVR: support vector regression; XGBoost: extreme gradient boosting.

Across the 6 target-horizon cells, statistical models won 6 of 6, classical ML models won 0 of 6, and primary neural models won 0 of 6. ARIMA won 5 cells and ETS won 1. The comparisons are descriptive and conditional on a frozen protocol and short official series.

For ASPH, ARIMA achieved an RMSE of 0.000178, an MAE of 0.000155, and a MASE of 0.031 at h=1; an RMSE of 0.000578, an MAE of 0.000512, and a MASE of 0.101 at h=2; and an RMSE of 0.004324, an MAE of 0.003877, and a MASE of 0.754 at h=5.

For MTC, ARIMA achieved an RMSE of 0.000400, an MAE of 0.000305, and a MASE of 0.024 at h=1, and an RMSE of 0.001479, an MAE of 0.001166, and a MASE of 0.090 at h=2. ETS won at h=5 with an RMSE of 0.006991, an MAE of 0.006203, and a MASE of 0.473.

### Predictive-Interval Coverage

Predictive intervals were frequently undercovered relative to nominal levels. Table 2 and Figure 5 report all 6 target-horizon cells and keep 2 descriptive estimands separate: the unweighted mean of model-level empirical coverage gives each interval-capable specification equal weight, whereas the pooled valid model-forecast coverage weights specifications by their available intervals. Unequal availability, particularly for rolling conformal ML intervals, means these quantities answer different questions. No inferential claims were made based on coverage proportions.

**Table 2.** Predictive-interval coverage across all target-horizon cells.^a^

Target	Horizon	Forecast, n	Nominal, %	Models, n	Valid intervals, n (range per specification)	Model mean	Pooled	Winner	Winner mean interval width
ASPH^b^	1	9	80	12	66 (2‐9)	0.690	0.545	ARIMA^c^: 1.000	0.000766
ASPH	1	9	95	12	66 (2‐9)	0.755	0.652	ARIMA: 1.000	0.001171
ASPH	2	8	80	6	48 (8‐8)	0.438	0.438	ARIMA: 1.000	0.002129
ASPH	2	8	95	6	48 (8‐8)	0.438	0.438	ARIMA: 1.000	0.003256
ASPH	5	5	80	6	30 (5‐5)	0.233	0.233	ARIMA: 0.400	0.008128
ASPH	5	5	95	6	30 (5‐5)	0.367	0.367	ARIMA: 1.000	0.012431
MTC^d^	1	18	80	12	174 (11‐18)	0.639	0.592	ARIMA: 0.500	0.000552
MTC	1	18	95	12	174 (11‐18)	0.708	0.678	ARIMA: 0.667	0.000844
MTC	2	17	80	12	156 (9‐17)	0.596	0.500	ARIMA: 0.471	0.001659
MTC	2	17	95	12	156 (9‐17)	0.679	0.609	ARIMA: 0.529	0.002537
MTC	5	14	80	12	102 (3‐14)	0.589	0.324	ETS^e^: 0.000	0.001744
MTC	5	14	95	12	102 (3‐14)	0.661	0.441	ETS: 0.000	0.001985

a Model mean is the unweighted mean of specification-level empirical coverage proportions. Pooled coverage is the proportion of all valid model-forecast intervals. The valid-interval field reports the total number of valid intervals and the range of n per specification. Neural models are excluded because no frozen method for constructing neural predictive-intervals is available. ASPH results at h=5 are extremely unstable because the forecast n is only 5. Winner mean interval width is the average difference between the upper and lower bounds across valid forecasts. Widths are reported in the target’s native units: proportion units for ASPH and millimoles per liter for MTC.

b ASPH: age-standardized prevalence of hypertension.

c ARIMA: autoregressive integrated moving average.

d MTC: mean total cholesterol.

e ETS: error-trend-seasonal state space.

**Figure 5.**
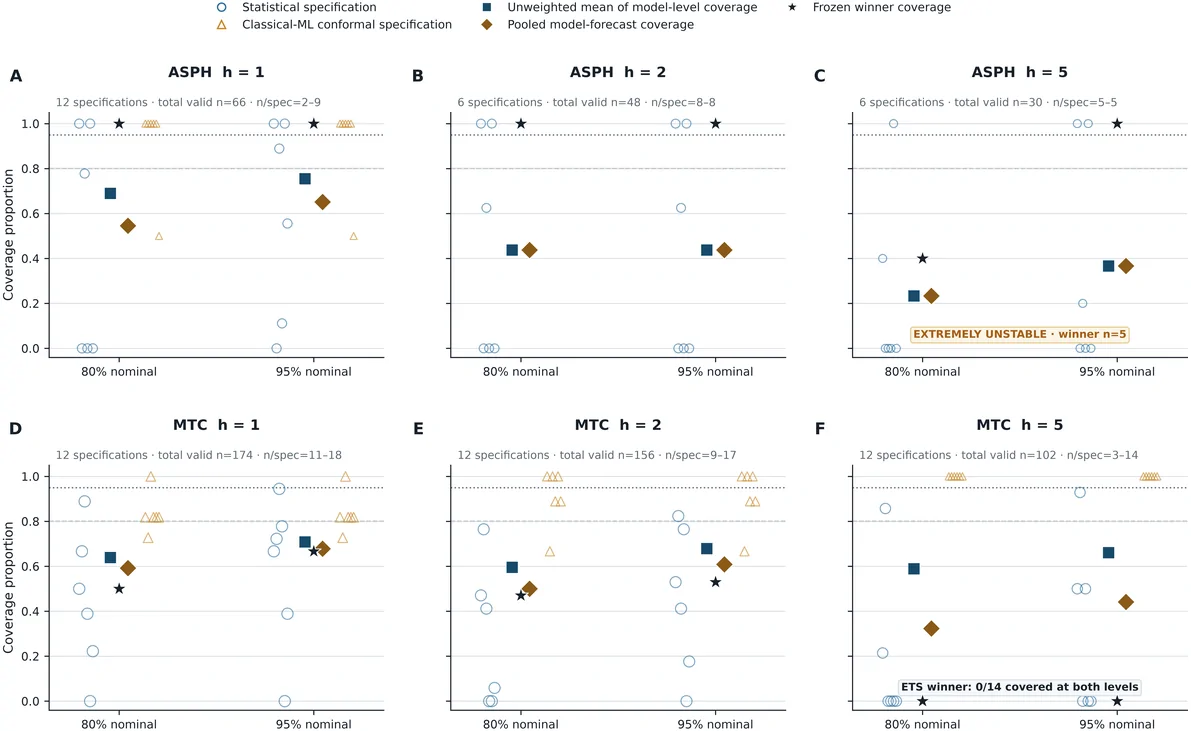
Empirical predictive-interval coverage by interval-capable model specification. Points show model-level coverage at nominal 80% and 95%, with statistical and classical-machine-learning (ML) interval methods distinguished, and the valid n is displayed. Reference lines mark the nominal levels; neural models are excluded because no frozen neural predictive-interval method exists. (A) Age-standardized prevalence of hypertension (ASPH) at the 1-year forecasting horizon (h=1, n=9); (B) ASPH at the 2-year forecasting horizon (h=2, n=8); (C) ASPH at the 5-year forecasting horizon (h=5, n=5); (D) mean total cholesterol (MTC) at the 1-year forecasting horizon (h=1, n=18); (E) MTC at the 2-year forecasting horizon (h=2, n=17); and (F) MTC at the 5-year forecasting horizon (h=5, n=14). ETS: error-trend-seasonal state space.

### R-Squared Diagnostic

Among the 114 primary specification-by-cell rows, 39 had negative diagnostic *R*², and none had undefined *R*²: 6 for ASPH h=1, 13 for ASPH h=2, 19 for ASPH h=5, 0 for MTC h=1, 0 for MTC h=2, and 1 for MTC h=5. The winning ASPH h=5 ARIMA specification had *R*²=−3.1898. A negative value can occur when the squared forecast error exceeds the constant-mean variance reference over the evaluation window; low target variability and a sample size of 5 make this statistic especially unstable. *R*² was used for diagnostics only and was not used for primary ranking.

### Attention Ablation

Removing attention produced lower RMSE than compact attention in 5 of 6 target-horizon cells. Compact attention was marginally better only for MTC h=1. Statistically significant attention differences occurred for ASPH h=1 and h=2; MTC nondetections were not interpreted as equivalence. The ASPH h=5 results were descriptive only because n=5 and the Diebold-Mariano test was disabled.

### Capacity Ablation

The compact, 857-parameter attention architecture had lower RMSE than the 22,369-parameter Original23K model in 5 of 6 cells. Original23K was better only for ASPH h=1. Statistically significant capacity differences were detected for ASPH h=1 and h=2; MTC nondetections were not interpreted as equivalence, and ASPH h=5 remained descriptive only (Figure 6).

**Figure 6.**
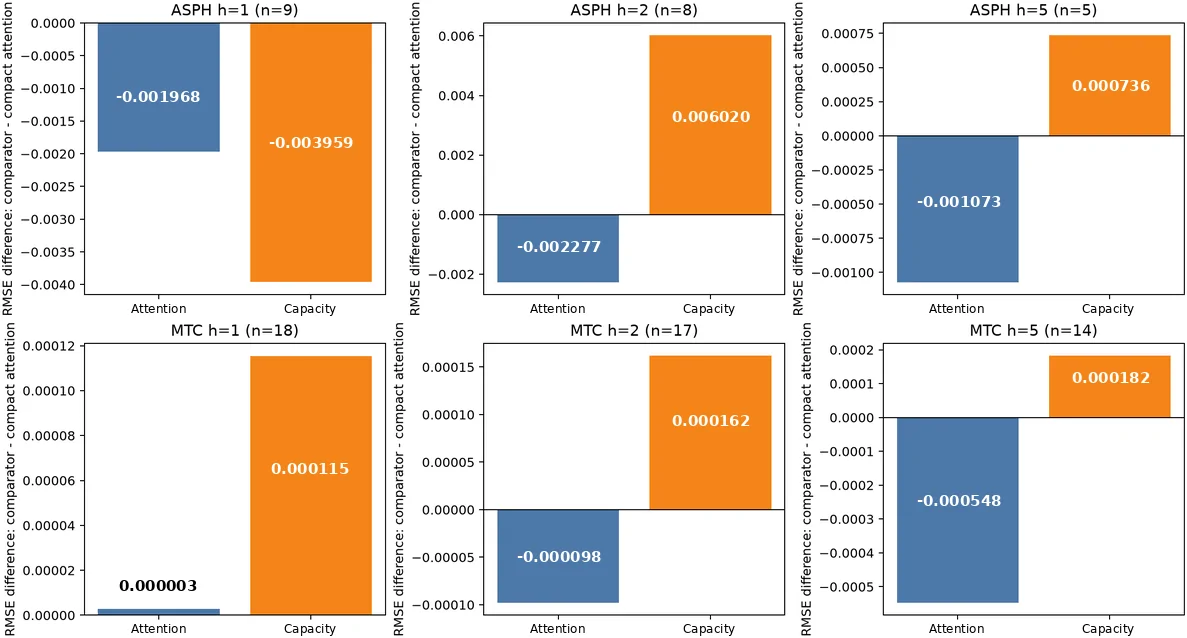
Prespecified neural attention and capacity ablations. The figure compares compact attention with no attention and with the Original23K attention architecture using the finalized evaluation results. Negative comparison-minus-compact root mean square error (RMSE) differences favor the comparison model. Age-standardized prevalence of hypertension (ASPH) h=5 is descriptive only (n=5), and neural seed dispersion is not a measure of predictive uncertainty. MTC: mean total cholesterol.

### Statistical Comparisons

Table 3 reports the 2 prespecified paired neural-ablation families. Where mathematically valid, the table includes paired-bootstrap intervals, HLN-corrected Diebold-Mariano statistics, and Holm-adjusted *P* values. Formal differences were detected for the attention and capacity contrasts at ASPH h=1 and h=2. The direction differed for capacity: Original23K was better at ASPH h=1, whereas the compact architecture was better at ASPH h=2.

**Table 3.** Prespecified neural attention and capacity ablation comparisons^a^.

Target	Horizon	Forecast, n	Contrast	ΔRMSE^b^ (bootstrap 95% CI)	DM^c^-HLN^d^	Holm-adjusted *P* value	Interpretation
ASPH^e^	1	9	Attention	–0.001968 (–0.002162 to –0.001791)	–14.548	<.001	Detected difference
ASPH	2	8	Attention	–0.002277 (–0.003113 to –0.001665)	–13.240	<.001	Detected difference
ASPH	5	5	Attention	–0.001073 (Not computed)	Not computed	Not computed	Descriptive only (n=5)
MTC^f^	1	18	Attention	0.000003 (–0.000193 to 0.000198)	0.026	.98	No difference detected; limited power
MTC	2	17	Attention	–0.000098 (–0.000251 to –0.000021)	–2.059	.17	No difference detected; limited power
MTC	5	14	Attention	–0.000548 (–0.001024 to 0.000034)	–0.890	.78	No difference detected; limited power
ASPH	1	9	Capacity	–0.003959 (–0.004319 to –0.003539)	–10.483	<.001	Detected difference
ASPH	2	8	Capacity	0.006020 (0.005386 to 0.006929)	13.824	<.001	Detected difference
ASPH	5	5	Capacity	0.000736 (Not computed)	Not computed	Not computed	Descriptive only (n=5)
MTC	1	18	Capacity	0.000115 (–0.000207 to 0.000340)	0.780	>.99	No difference detected; limited power
MTC	2	17	Capacity	0.000162 (–0.001227 to 0.001680)	0.178	>.99	No difference detected; limited power
MTC	5	14	Capacity	0.000182 (–0.001312 to 0.002135)	0.198	>.99	No difference detected; limited power

a ΔRMSE is calculated as the comparison model’s RMSE minus the compact-attention model’s RMSE; negative values favor the comparison model: no-attention or Original23K. Reported bootstrap 95% CIs for the 10 inferentially valid cells were calculated using 5000 paired repetitions. Holm-adjusted *P* values were calculated separately within each prespecified ablation family. ASPH at h=5 (n=5) was descriptive only; its bootstrap CI, DM-HLN statistic, and *P* value are reported as “Not computed” because formal inference was disabled a priori. Nondetection is not an evidence of equivalence.

b RMSE: root mean square error.

c DM: Diebold-Mariano.

d HLN: Harvey-Leybourne-Newbold.

e ASPH: age-standardized prevalence of hypertension.

f MTC: mean total cholesterol.

No difference was detected for any MTC ablation contrast under the prespecified procedure; this is a statement of limited evidence, not equivalence. ASPH h=5 had only 5 paired forecasts, so the Diebold-Mariano test was disabled a priori, and the comparison remained descriptive. Primary cross-model rankings were descriptive because no prespecified general model-comparison family existed; no post hoc winner-vs-rest tests were created.

Figure 3 summarizes initialization variability across the 7 primary neural architectures and 6 target-horizon cells.

### Uncertainty Interpretation

Winner-specific coverage illustrates the undercoverage. For MTC h=1 ARIMA, empirical coverage was 0.500 at nominal 80% and 0.667 at nominal 95%; for MTC h=2 ARIMA, it was 0.471 and 0.529, respectively. For MTC h=5 ETS, coverage was 0 at both levels across 14 forecasts. In that cell, the unweighted mean of model-level coverage was 0.589 at 80% and 0.661 at 95%, whereas pooled model-forecast coverage was 0.324 and 0.441, respectively. These descriptive proportions are substantially below nominal for many specifications.

Neural seed standard deviation is conditional on a fixed architecture, representation, lookback, epoch budget, and training sample. It answers how much the fitted function changes with initialization; it does not quantify whether a future observation will fall within a calibrated interval. Therefore, seed averaging does not repair empirical undercoverage, and the 2 uncertainty quantities are not combined a single confidence statement.

### Computational Cost

The benchmark completed all 7242 planned output-producing fits. There were 7243 attempted executions because one initial parallel neural fit encountered a MemoryError; the unchanged fit was rerun successfully after limiting BLAS/OpenMP threading, leaving 0 unresolved failures. Overall wall-clock time was 1903.845 seconds (31 minutes 43.845 seconds), cumulative per-fit elapsed time was 7445.070 seconds, and cumulative estimator-fitting time was 6797.499 seconds. The cumulative sums include parallel work and are not alternative wall-clock durations. Detailed family-level timing is reported in Table S3 of Multimedia Appendix 1.

Computational cost is relevant when the accuracy gain is small or not statistically detected. The CPU-only execution used an Intel Core Ultra 9 185H processor with 16 physical and 22 logical cores, approximately 33.7 GB of RAM, no GPU, and 18 neural worker processes, each with one computational thread. Timing may differ on other hardware.

## Discussion

### Principal Findings

The central finding is that statistical models produced the lowest RMSE in all 6 target-horizon cells: ARIMA in 5 and ETS in 1. Classical ML models and primary neural models won no cells. This does not establish a universal hierarchy; it shows that, for these 2 short official annual series under the frozen design, added flexibility did not improve the primary point-forecast ranking.

The target-specific results must be interpreted in light of different units, source populations, and evaluation counts. ASPH has 30 observations and only 9, 8, and 5 forecast targets across h=1, h=2, and h=5; MTC has 39 observations and 18, 17, and 14 targets. Longer horizons expose forecasts to greater trend error, while ASPH at h=5 is especially unstable because only 5 observations contribute to each summary.

Fold-local first-differencing and linear detrending defined the prespecified, leakage-controlled specifications. In the descriptive primary ranking, first-difference classical-ML specifications ranked 3rd to 12th out of 19, whereas detrended classical-ML specifications ranked 12th to 18th. ARIMA ranked first in 5 cells, and ETS ranked first in one and second in 4 others. Naive ranked 17th to 19th in every MTC cell, while drift and theta each ranked 11th to 15th in 5 of 6 cells. These cell-specific results support retaining statistical benchmarks without implying that all simple baselines performed comparably or that the ranking is universal.

The neural results also argue against a monotonic complexity-to-accuracy relationship. Ten-seed evaluation made initialization sensitivity visible, yet removing attention reduced RMSE in 5 of 6 cells, and the compact architecture outperformed Original23K in 5 of 6. Recent forecasting work has likewise shown that lightweight models can remain competitive with much larger architectures on some benchmarks [24]. This study does not establish a general law favoring small models; architectural additions should earn their complexity through repeated out-of-sample evidence.

The prespecified paired analyses support a narrow conclusion. Attention and capacity differences were detected for ASPH h=1 and h=2, but no MTC contrast was detected, and nondetection is not evidence of equivalence. ASPH h=5 was only descriptive because n=5. Because no broad development-only model-comparison family was prespecified, primary cross-model rankings remain descriptive, and no post hoc winner-vs-rest inference was performed.

Point accuracy and uncertainty calibration answer different questions. Several interval methods substantially undercovered, including zero coverage at both nominal levels for the MTC h=5 ETS winner. Model-level mean and pooled coverage also differed when interval availability was unequal. Negative diagnostic *R*² values, including −3.1898 for the winning ASPH h=5 ARIMA model, reflect error relative to a low-variance, constant-mean reference over a tiny window and do not contradict the RMSE ranking. *R*² was used for diagnostic only.

### Comparison With Prior Work

The findings are consistent with the broader empirical forecasting literature, in which simple statistical methods and carefully designed combinations remain difficult to beat across heterogeneous collections of series [6-9]. Large forecasting competitions have repeatedly shown that the strongest approach depends on data characteristics, horizon, and evaluation design rather than on a single universally superior algorithm. Our results extend that lesson to 2 short annual health series: low-complexity baselines remain essential comparators even when modern ML and neural models are included.

At the same time, deep forecasting has advanced substantially through recurrent probabilistic models, basis-expansion architectures, multi-horizon attention, transformers, and pretrained time-series foundation models [5,15-23]. These methods can exploit large cross-series corpora and transferable representations—capabilities that are fundamentally different from those required for learning annual sequences containing 30 or 39 values. The present results should not be read as evidence against modern forecasting architectures; they show that large-scale success does not remove the need for task-matched baselines and leakage-controlled local evaluation.

Recent work highlights 2 themes relevant beyond the present design. Parameter-efficient models can be competitive in long-horizon forecasting, as illustrated by SparseTSF [24], and future test periods can differ from historical training data, motivating out-of-distribution research [14]. These literatures motivate future external validation; the current benchmark did not conduct a separate recent-period or regime analysis.

Health care forecasting benchmarks have demonstrated the value of standardized evaluation for high-frequency clinical time series and longitudinal patient records [25-27]. This study addresses a different use case: annual population-level risk-factor forecasting under extreme sample scarcity. By combining official-source reconstruction, rolling-origin evaluation, fixed protocol choices, model-family comparison, and explicit separation of interval coverage from seed sensitivity, the benchmark makes that setting’s limitations visible, rather than obscuring them behind a single headline model.

### Practical Implications

For surveillance and planning teams, the results support prespecifying candidate models separately for each indicator and horizon, retaining simple statistical baselines, and evaluating interval calibration alongside point error. Short-horizon forecasts may support surveillance review, whereas longer-horizon estimates require greater emphasis on uncertainty and extrapolation risk. These findings do not establish clinical utility, resource savings, or transportability; operational use would require prospective data vintages, ongoing monitoring, external validation, and decision-specific assessment.

### Future Directions

Future work should evaluate the protocol on longer and geographically diverse series, compare alternative interval methods, and test whether modern pretrained forecasters provide reliable gains when only a short target series is available. Extensions should preserve leakage-controlled repeated origins, direct horizon-specific targets, and prespecified choices, and should examine women, other countries, and other risk factors. Any new source should have a citable data lineage and an independent external evaluation rather than inheriting the present ranking as a universal conclusion.

### Strengths

The study has several strengths: fully reconstructed official-source provenance; exclusion rather than extension of unsupported years; a common expanding-window protocol with H=21; fixed L=3 without evaluation-driven lookback tuning; fold-local transformations; 10 fixed neural seeds; prespecified attention and capacity ablations; complete forecasting metrics; all-6-cell interval reporting; reconciled failure and timing accounts; and public reproducibility materials.

### Limitations

First, the source series are short: ASPH contains 30 annual observations and MTC 39. Their evaluation samples are only 9/8/5 forecasts for ASPH and 18/17/14 for MTC at h=1/h=2/h=5. In particular, ASPH h=5 has n=5, making comparative summaries, coverage, and diagnostic *R*² extremely unstable; the Diebold-Mariano test was disabled for that cell.

Second, the outcomes represent different source populations: ASPH covers men aged 30 to 79 years, whereas MTC covers men aged 18 years and older. Only data from China and only the male population were analyzed, so the findings should not be generalized automatically to women, other countries, other risk factors, or higher-frequency data.

Third, formal inference was restricted to 2 prespecified neural ablation families. Nondetection, particularly for MTC, does not constitute evidence of equivalence, and no prespecified broad cross-model testing family existed. Longer series or prespecified replications are required for stronger inference.

Fourth, empirical predictive-interval coverage was often substantially below nominal levels. Unequal valid interval counts mean that the unweighted mean of model-level coverage and pooled model-forecast coverage answer different questions. No frozen neural predictive-interval method was available, and neural seed dispersion cannot substitute for one.

Fifth, the primary ML and neural lookback was fixed at L=3 for sample adequacy, and the finalized benchmark did not evaluate alternative lookbacks. The architecture set was broad but not exhaustive, and CPU-only timing may not represent execution on other hardware.

Sixth, both targets are retrospective, model-based annual NCD-RisC estimates rather than historical real-time data vintages. The design controls for leakage within the published series but cannot reproduce the information-release process of a prospective forecast. In addition, explicit dataset-level permission to redistribute the NCD-RisC numerical files was not found, so readers must retrieve the source files directly.

### Conclusions

Across the fully sourced China-male ASPH and MTC series, statistical models produced the lowest point-forecast RMSE in all 6 evaluated target-horizon cells: ARIMA in 5 of 6 and ETS in 1 of 6, while classical ML and primary neural models won 0 of 6. This result is conditional on 2 short, retrospective annual estimate series and does not establish universal superiority. Attention and greater capacity provided no consistent advantage, and strong point prediction did not guarantee calibrated intervals. Short annual population-health series require strong simple baselines, leakage-controlled evaluation, and transparent uncertainty reporting.

## Supplementary material

10.2196/99686 Multimedia Appendix 1 Complete primary metrics, prespecified neural ablations, initialization variability, predictive-interval coverage, and computational reproducibility.

## Abbreviations

ARIMA: autoregressive integrated moving average
ASPH: age-standardized prevalence of hypertension
BiLSTM: bidirectional long short-term memory
CNN: convolutional neural network
ETS: error-trend-seasonal state space
GHO: Global Health Observatory
GRU: gated recurrent unit
HLN: Harvey-Leybourne-Newbold
LSTM: long short-term memory
MAE: mean absolute error
MASE: mean absolute scaled error
ML: machine learning
MTC: mean total cholesterol
NCD-RisC: NCD Risk Factor Collaboration
RMSE: root mean square error
RMSSE: root mean squared scaled error
SVR: support vector regression
WHO: World Health Organization
XGBoost: extreme gradient boosting
