# Supplementary material for: Horizon-Aware Forecasting of Hypertension Prevalence and Mean Total Cholesterol in Chinese Men: Retrospective Leakage-Controlled Benchmark Study Comparing Statistical, Machine-Learning, and Neural Models
Source: JMIR Med Inform. 2026 Nov 11;14:e99686. doi: 10.2196/99686 (PMC13754258; doi:10.2196/99686)
Supplement: Supplementary file 1 [file medinform-v14-e99686-s001.docx]

Multimedia Appendix 1

Complete Primary Metrics, Prespecified Neural Ablations, and Computational Reproducibility

Supplement to: Horizon-Aware Forecasting of Hypertension Prevalence and Mean Total Cholesterol in Chinese Men: A Leakage-Controlled Benchmark Across Statistical, Machine-Learning, and Neural Models

## Complete Primary-Specification Forecasting Metrics

Multimedia Appendix Table 1. Complete primary-specification forecasting metrics

| **Target** | **h** | **n** | **Family** | **Specification** | **RMSE** | **MAE** | **MASE** | **RMSSE** | **sMAPE, %** | **Direction, %** | **Mean error** | **Median error** | **R²** |
| --- | --- | --- | --- | --- | --- | --- | --- | --- | --- | --- | --- | --- | --- |
| ASPH | 1 | 9 | Statistical | Naive | 0.001476 | 0.001448 | 0.300 | 0.258 | 0.469 | 0.0 | -0.001448 | -0.001502 | 0.868 |
| ASPH | 1 | 9 | Statistical | Drift | 0.005935 | 0.005907 | 1.208 | 1.024 | 1.895 | 0.0 | -0.005907 | -0.006033 | -1.137 |
| ASPH | 1 | 9 | Statistical | Linear Trend | 0.029490 | 0.029173 | 6.068 | 5.203 | 9.038 | 0.0 | -0.029173 | -0.030512 | -51.762 |
| ASPH | 1 | 9 | Statistical | ETS | 0.000552 | 0.000345 | 0.065 | 0.089 | 0.110 | 88.9 | -0.000338 | -0.000100 | 0.982 |
| ASPH | 1 | 9 | Statistical | ARIMA | 0.000178 | 0.000155 | 0.031 | 0.030 | 0.050 | 100.0 | 0.000077 | 0.000151 | 0.998 |
| ASPH | 1 | 9 | Statistical | Theta | 0.003677 | 0.003661 | 0.750 | 0.636 | 1.179 | 0.0 | -0.003661 | -0.003684 | 0.180 |
| ASPH | 1 | 9 | Classical ML | SVR—first difference | 0.000372 | 0.000242 | 0.048 | 0.062 | 0.078 | 100.0 | -0.000169 | -0.000008 | 0.992 |
| ASPH | 1 | 9 | Classical ML | Random Forest—first difference | 0.001100 | 0.000753 | 0.144 | 0.181 | 0.241 | 77.8 | -0.000682 | -0.000039 | 0.927 |
| ASPH | 1 | 9 | Classical ML | XGBoost—first difference | 0.002300 | 0.001920 | 0.379 | 0.386 | 0.615 | 33.3 | -0.001920 | -0.002313 | 0.679 |
| ASPH | 1 | 9 | Classical ML | SVR—detrended | 0.009761 | 0.008517 | 1.696 | 1.651 | 2.697 | 0.0 | -0.008517 | -0.010028 | -4.780 |
| ASPH | 1 | 9 | Classical ML | Random Forest—detrended | 0.013637 | 0.013286 | 2.724 | 2.359 | 4.209 | 0.0 | -0.013286 | -0.013266 | -10.283 |
| ASPH | 1 | 9 | Classical ML | XGBoost—detrended | 0.019364 | 0.018683 | 3.851 | 3.373 | 5.868 | 0.0 | -0.018683 | -0.019051 | -21.748 |
| ASPH | 1 | 9 | Primary neural | LSTM | 0.001977 | 0.001846 | 0.370 | 0.335 | 0.594 | 22.2 | -0.001805 | -0.001906 | 0.763 |
| ASPH | 1 | 9 | Primary neural | GRU | 0.000734 | 0.000555 | 0.108 | 0.120 | 0.178 | 88.9 | -0.000365 | -0.000099 | 0.967 |
| ASPH | 1 | 9 | Primary neural | BiLSTM | 0.000237 | 0.000213 | 0.044 | 0.041 | 0.069 | 100.0 | 0.000104 | 0.000219 | 0.997 |
| ASPH | 1 | 9 | Primary neural | Causal CNN | 0.000553 | 0.000368 | 0.071 | 0.090 | 0.118 | 88.9 | -0.000159 | 0.000021 | 0.981 |
| ASPH | 1 | 9 | Primary neural | CNN-LSTM | 0.000385 | 0.000320 | 0.064 | 0.064 | 0.103 | 88.9 | -0.000089 | 0.000139 | 0.991 |
| ASPH | 1 | 9 | Primary neural | CNN-GRU | 0.001298 | 0.001096 | 0.215 | 0.215 | 0.351 | 66.7 | -0.001062 | -0.000917 | 0.898 |
| ASPH | 1 | 9 | Primary neural | CNN-BiLSTM compact attention | 0.004175 | 0.004126 | 0.842 | 0.719 | 1.327 | 0.0 | -0.004126 | -0.004352 | -0.058 |
| ASPH | 2 | 8 | Statistical | Naive | 0.003023 | 0.002997 | 0.610 | 0.523 | 0.970 | 0.0 | -0.002997 | -0.003054 | 0.303 |
| ASPH | 2 | 8 | Statistical | Drift | 0.012193 | 0.012143 | 2.450 | 2.086 | 3.866 | 0.0 | -0.012143 | -0.012363 | -10.334 |
| ASPH | 2 | 8 | Statistical | Linear Trend | 0.034862 | 0.034672 | 7.085 | 6.063 | 10.673 | 0.0 | -0.034672 | -0.035826 | -91.657 |
| ASPH | 2 | 8 | Statistical | ETS | 0.001405 | 0.000921 | 0.174 | 0.227 | 0.295 | 87.5 | -0.000921 | -0.000317 | 0.850 |
| ASPH | 2 | 8 | Statistical | ARIMA | 0.000578 | 0.000512 | 0.101 | 0.097 | 0.166 | 100.0 | 0.000305 | 0.000402 | 0.975 |
| ASPH | 2 | 8 | Statistical | Theta | 0.007571 | 0.007547 | 1.526 | 1.298 | 2.422 | 0.0 | -0.007547 | -0.007581 | -3.369 |
| ASPH | 2 | 8 | Classical ML | SVR—first difference | 0.005335 | 0.003628 | 0.687 | 0.870 | 1.151 | 62.5 | -0.003532 | -0.001774 | -1.170 |
| ASPH | 2 | 8 | Classical ML | Random Forest—first difference | 0.003965 | 0.003306 | 0.648 | 0.655 | 1.060 | 75.0 | -0.003306 | -0.002458 | -0.199 |
| ASPH | 2 | 8 | Classical ML | XGBoost—first difference | 0.003509 | 0.002708 | 0.519 | 0.574 | 0.865 | 62.5 | -0.002708 | -0.001978 | 0.061 |
| ASPH | 2 | 8 | Classical ML | SVR—detrended | 0.028562 | 0.028355 | 5.723 | 4.889 | 8.797 | 0.0 | -0.028355 | -0.029522 | -61.193 |
| ASPH | 2 | 8 | Classical ML | Random Forest—detrended | 0.028574 | 0.028547 | 5.802 | 4.934 | 8.869 | 0.0 | -0.028547 | -0.028734 | -61.244 |
| ASPH | 2 | 8 | Classical ML | XGBoost—detrended | 0.032302 | 0.032207 | 6.559 | 5.593 | 9.950 | 0.0 | -0.032207 | -0.032853 | -78.548 |
| ASPH | 2 | 8 | Primary neural | Causal CNN | 0.002360 | 0.001829 | 0.359 | 0.387 | 0.590 | 87.5 | 0.000159 | 0.000997 | 0.576 |
| ASPH | 2 | 8 | Primary neural | CNN-LSTM | 0.003694 | 0.002955 | 0.571 | 0.603 | 0.946 | 62.5 | -0.002955 | -0.002268 | -0.040 |
| ASPH | 2 | 8 | Primary neural | CNN-GRU | 0.003034 | 0.002130 | 0.404 | 0.493 | 0.680 | 75.0 | -0.002061 | -0.001406 | 0.298 |
| ASPH | 2 | 8 | Primary neural | CNN-BiLSTM compact attention | 0.007125 | 0.007000 | 1.403 | 1.208 | 2.244 | 0.0 | -0.007000 | -0.006796 | -2.871 |
| ASPH | 2 | 8 | Primary neural | LSTM | 0.031163 | 0.030937 | 6.323 | 5.418 | 9.578 | 0.0 | -0.030937 | -0.032916 | -73.035 |
| ASPH | 2 | 8 | Primary neural | GRU | 0.026202 | 0.025824 | 5.295 | 4.571 | 8.059 | 0.0 | -0.025824 | -0.028336 | -51.339 |
| ASPH | 2 | 8 | Primary neural | BiLSTM | 0.026937 | 0.026643 | 5.453 | 4.690 | 8.304 | 0.0 | -0.026643 | -0.028764 | -54.317 |
| ASPH | 5 | 5 | Statistical | Naive | 0.007755 | 0.007746 | 1.506 | 1.299 | 2.506 | 0.0 | -0.007746 | -0.007939 | -12.480 |
| ASPH | 5 | 5 | Statistical | Drift | 0.032580 | 0.032524 | 6.298 | 5.430 | 10.110 | 0.0 | -0.032524 | -0.032853 | -236.892 |
| ASPH | 5 | 5 | Statistical | Linear Trend | 0.052233 | 0.052199 | 10.153 | 8.754 | 15.754 | 0.0 | -0.052199 | -0.052807 | -610.472 |
| ASPH | 5 | 5 | Statistical | ETS | 0.005259 | 0.004105 | 0.768 | 0.850 | 1.323 | 80.0 | -0.004105 | -0.002840 | -5.199 |
| ASPH | 5 | 5 | Statistical | ARIMA | 0.004324 | 0.003877 | 0.754 | 0.722 | 1.280 | 100.0 | 0.003341 | 0.004369 | -3.190 |
| ASPH | 5 | 5 | Statistical | Theta | 0.020110 | 0.020092 | 3.895 | 3.356 | 6.370 | 0.0 | -0.020092 | -0.020492 | -89.638 |
| ASPH | 5 | 5 | Classical ML | SVR—first difference | 0.022428 | 0.021076 | 4.043 | 3.703 | 6.634 | 0.0 | -0.021076 | -0.024794 | -111.737 |
| ASPH | 5 | 5 | Classical ML | Random Forest—first difference | 0.014909 | 0.014428 | 2.768 | 2.459 | 4.602 | 0.0 | -0.014428 | -0.015929 | -48.819 |
| ASPH | 5 | 5 | Classical ML | XGBoost—first difference | 0.015639 | 0.014855 | 2.840 | 2.569 | 4.729 | 0.0 | -0.014855 | -0.015870 | -53.819 |
| ASPH | 5 | 5 | Classical ML | SVR—detrended | 0.056980 | 0.056871 | 11.075 | 9.565 | 17.042 | 0.0 | -0.056871 | -0.056191 | -726.662 |
| ASPH | 5 | 5 | Classical ML | Random Forest—detrended | 0.059496 | 0.059448 | 11.565 | 9.974 | 17.747 | 0.0 | -0.059448 | -0.060137 | -792.337 |
| ASPH | 5 | 5 | Classical ML | XGBoost—detrended | 0.059854 | 0.059814 | 11.635 | 10.032 | 17.847 | 0.0 | -0.059814 | -0.060206 | -801.935 |
| ASPH | 5 | 5 | Primary neural | LSTM | 0.059371 | 0.059344 | 11.538 | 9.945 | 17.718 | 0.0 | -0.059344 | -0.060174 | -789.025 |
| ASPH | 5 | 5 | Primary neural | GRU | 0.054443 | 0.054413 | 10.561 | 9.100 | 16.363 | 0.0 | -0.054413 | -0.053542 | -663.315 |
| ASPH | 5 | 5 | Primary neural | BiLSTM | 0.060808 | 0.060762 | 11.820 | 10.193 | 18.104 | 0.0 | -0.060762 | -0.061393 | -827.716 |
| ASPH | 5 | 5 | Primary neural | Causal CNN | 0.059721 | 0.059685 | 11.607 | 10.008 | 17.811 | 0.0 | -0.059685 | -0.060934 | -798.370 |
| ASPH | 5 | 5 | Primary neural | CNN-LSTM | 0.058090 | 0.058074 | 11.283 | 9.722 | 17.372 | 0.0 | -0.058074 | -0.058232 | -755.282 |
| ASPH | 5 | 5 | Primary neural | CNN-GRU | 0.052688 | 0.052597 | 10.195 | 8.791 | 15.855 | 0.0 | -0.052597 | -0.052997 | -621.166 |
| ASPH | 5 | 5 | Primary neural | CNN-BiLSTM compact attention | 0.057665 | 0.057620 | 11.208 | 9.666 | 17.248 | 0.0 | -0.057620 | -0.057867 | -744.257 |
| MTC | 1 | 18 | Statistical | Naive | 0.009950 | 0.009867 | 0.768 | 0.754 | 0.221 | 0.0 | 0.009867 | 0.010021 | 0.966 |
| MTC | 1 | 18 | Statistical | Drift | 0.003288 | 0.002993 | 0.232 | 0.247 | 0.067 | 100.0 | -0.002993 | -0.002691 | 0.996 |
| MTC | 1 | 18 | Statistical | Linear Trend | 0.021857 | 0.021596 | 1.676 | 1.647 | 0.481 | 100.0 | -0.021596 | -0.020791 | 0.835 |
| MTC | 1 | 18 | Statistical | ETS | 0.000528 | 0.000454 | 0.035 | 0.040 | 0.010 | 100.0 | -0.000013 | -0.000178 | 1.000 |
| MTC | 1 | 18 | Statistical | ARIMA | 0.000400 | 0.000305 | 0.024 | 0.030 | 0.007 | 100.0 | -0.000136 | -0.000085 | 1.000 |
| MTC | 1 | 18 | Statistical | Theta | 0.003694 | 0.003437 | 0.268 | 0.281 | 0.077 | 100.0 | 0.003437 | 0.003731 | 0.995 |
| MTC | 1 | 18 | Classical ML | SVR—first difference | 0.000529 | 0.000436 | 0.034 | 0.039 | 0.010 | 100.0 | -0.000041 | -0.000031 | 1.000 |
| MTC | 1 | 18 | Classical ML | Random Forest—first difference | 0.001007 | 0.000791 | 0.061 | 0.076 | 0.018 | 100.0 | 0.000150 | -0.000082 | 1.000 |
| MTC | 1 | 18 | Classical ML | XGBoost—first difference | 0.000866 | 0.000690 | 0.053 | 0.065 | 0.015 | 100.0 | -0.000059 | -0.000116 | 1.000 |
| MTC | 1 | 18 | Classical ML | SVR—detrended | 0.009078 | 0.005449 | 0.407 | 0.652 | 0.123 | 100.0 | -0.005406 | -0.001501 | 0.972 |
| MTC | 1 | 18 | Classical ML | Random Forest—detrended | 0.009399 | 0.008613 | 0.664 | 0.698 | 0.193 | 100.0 | -0.008613 | -0.006845 | 0.970 |
| MTC | 1 | 18 | Classical ML | XGBoost—detrended | 0.010620 | 0.009344 | 0.716 | 0.781 | 0.209 | 100.0 | -0.009344 | -0.007278 | 0.961 |
| MTC | 1 | 18 | Primary neural | LSTM | 0.000967 | 0.000776 | 0.060 | 0.073 | 0.017 | 100.0 | 0.000078 | -0.000196 | 1.000 |
| MTC | 1 | 18 | Primary neural | GRU | 0.001015 | 0.000766 | 0.059 | 0.076 | 0.017 | 100.0 | 0.000325 | -0.000077 | 1.000 |
| MTC | 1 | 18 | Primary neural | BiLSTM | 0.001266 | 0.000903 | 0.069 | 0.092 | 0.020 | 100.0 | -0.000809 | -0.000490 | 0.999 |
| MTC | 1 | 18 | Primary neural | Causal CNN | 0.000870 | 0.000775 | 0.061 | 0.066 | 0.017 | 100.0 | 0.000030 | -0.000416 | 1.000 |
| MTC | 1 | 18 | Primary neural | CNN-LSTM | 0.001015 | 0.000809 | 0.062 | 0.076 | 0.018 | 100.0 | -0.000039 | -0.000138 | 1.000 |
| MTC | 1 | 18 | Primary neural | CNN-GRU | 0.000929 | 0.000752 | 0.058 | 0.070 | 0.017 | 100.0 | 0.000043 | -0.000212 | 1.000 |
| MTC | 1 | 18 | Primary neural | CNN-BiLSTM compact attention | 0.001101 | 0.000894 | 0.069 | 0.082 | 0.020 | 100.0 | -0.000056 | -0.000274 | 1.000 |
| MTC | 2 | 17 | Statistical | Naive | 0.020114 | 0.019973 | 1.550 | 1.520 | 0.446 | 0.0 | 0.019973 | 0.020278 | 0.844 |
| MTC | 2 | 17 | Statistical | Drift | 0.006364 | 0.005833 | 0.450 | 0.476 | 0.130 | 100.0 | -0.005833 | -0.005460 | 0.984 |
| MTC | 2 | 17 | Statistical | Linear Trend | 0.024762 | 0.024402 | 1.886 | 1.856 | 0.543 | 100.0 | -0.024402 | -0.022575 | 0.764 |
| MTC | 2 | 17 | Statistical | ETS | 0.001553 | 0.001340 | 0.104 | 0.116 | 0.030 | 100.0 | 0.000022 | -0.000786 | 0.999 |
| MTC | 2 | 17 | Statistical | ARIMA | 0.001479 | 0.001166 | 0.090 | 0.111 | 0.026 | 100.0 | -0.000629 | -0.000708 | 0.999 |
| MTC | 2 | 17 | Statistical | Theta | 0.007484 | 0.007054 | 0.549 | 0.567 | 0.157 | 100.0 | 0.007054 | 0.007416 | 0.978 |
| MTC | 2 | 17 | Classical ML | SVR—first difference | 0.002275 | 0.001406 | 0.106 | 0.163 | 0.032 | 100.0 | 0.000013 | 0.000240 | 0.998 |
| MTC | 2 | 17 | Classical ML | Random Forest—first difference | 0.002451 | 0.001808 | 0.138 | 0.180 | 0.041 | 100.0 | 0.000067 | -0.000395 | 0.998 |
| MTC | 2 | 17 | Classical ML | XGBoost—first difference | 0.002442 | 0.001984 | 0.153 | 0.182 | 0.044 | 100.0 | 0.000984 | 0.000926 | 0.998 |
| MTC | 2 | 17 | Classical ML | SVR—detrended | 0.015770 | 0.012470 | 0.948 | 1.153 | 0.279 | 100.0 | -0.012119 | -0.009832 | 0.904 |
| MTC | 2 | 17 | Classical ML | Random Forest—detrended | 0.017377 | 0.016494 | 1.268 | 1.289 | 0.368 | 100.0 | -0.016494 | -0.014178 | 0.884 |
| MTC | 2 | 17 | Classical ML | XGBoost—detrended | 0.018229 | 0.017148 | 1.318 | 1.352 | 0.383 | 100.0 | -0.017148 | -0.015164 | 0.872 |
| MTC | 2 | 17 | Primary neural | LSTM | 0.002811 | 0.002120 | 0.162 | 0.207 | 0.047 | 100.0 | 0.000150 | -0.000298 | 0.997 |
| MTC | 2 | 17 | Primary neural | GRU | 0.002771 | 0.002183 | 0.167 | 0.205 | 0.049 | 100.0 | 0.000778 | -0.000262 | 0.997 |
| MTC | 2 | 17 | Primary neural | BiLSTM | 0.005135 | 0.004467 | 0.344 | 0.382 | 0.100 | 100.0 | -0.004467 | -0.004006 | 0.990 |
| MTC | 2 | 17 | Primary neural | Causal CNN | 0.002603 | 0.002294 | 0.177 | 0.195 | 0.051 | 100.0 | 0.000431 | -0.000930 | 0.997 |
| MTC | 2 | 17 | Primary neural | CNN-LSTM | 0.002929 | 0.002270 | 0.174 | 0.216 | 0.051 | 100.0 | 0.000108 | -0.000390 | 0.997 |
| MTC | 2 | 17 | Primary neural | CNN-GRU | 0.002781 | 0.002124 | 0.163 | 0.204 | 0.048 | 100.0 | -0.000009 | -0.000412 | 0.997 |
| MTC | 2 | 17 | Primary neural | CNN-BiLSTM compact attention | 0.003064 | 0.002335 | 0.179 | 0.224 | 0.052 | 100.0 | -0.000792 | -0.000718 | 0.996 |
| MTC | 5 | 14 | Statistical | Naive | 0.051658 | 0.051485 | 3.955 | 3.864 | 1.151 | 0.0 | 0.051485 | 0.051998 | -0.576 |
| MTC | 5 | 14 | Statistical | Drift | 0.014348 | 0.013639 | 1.045 | 1.068 | 0.303 | 100.0 | -0.013639 | -0.013278 | 0.878 |
| MTC | 5 | 14 | Statistical | Linear Trend | 0.033292 | 0.032857 | 2.514 | 2.472 | 0.728 | 100.0 | -0.032857 | -0.030859 | 0.345 |
| MTC | 5 | 14 | Statistical | ETS | 0.006991 | 0.006203 | 0.473 | 0.516 | 0.138 | 100.0 | 0.000920 | -0.002791 | 0.971 |
| MTC | 5 | 14 | Statistical | ARIMA | 0.010457 | 0.008950 | 0.684 | 0.775 | 0.199 | 100.0 | -0.005526 | -0.006592 | 0.935 |
| MTC | 5 | 14 | Statistical | Theta | 0.019175 | 0.018711 | 1.440 | 1.438 | 0.416 | 100.0 | 0.018711 | 0.019178 | 0.783 |
| MTC | 5 | 14 | Classical ML | SVR—first difference | 0.009770 | 0.006312 | 0.470 | 0.697 | 0.141 | 100.0 | -0.003026 | 0.000308 | 0.944 |
| MTC | 5 | 14 | Classical ML | Random Forest—first difference | 0.007800 | 0.006563 | 0.499 | 0.572 | 0.146 | 100.0 | 0.001857 | 0.003886 | 0.964 |
| MTC | 5 | 14 | Classical ML | XGBoost—first difference | 0.008609 | 0.007916 | 0.609 | 0.643 | 0.176 | 100.0 | 0.005370 | 0.007135 | 0.956 |
| MTC | 5 | 14 | Classical ML | SVR—detrended | 0.025362 | 0.022903 | 1.749 | 1.873 | 0.508 | 100.0 | -0.022903 | -0.024219 | 0.620 |
| MTC | 5 | 14 | Classical ML | Random Forest—detrended | 0.040248 | 0.039749 | 3.042 | 2.989 | 0.880 | 100.0 | -0.039749 | -0.038063 | 0.043 |
| MTC | 5 | 14 | Classical ML | XGBoost—detrended | 0.038610 | 0.038264 | 2.930 | 2.871 | 0.847 | 100.0 | -0.038264 | -0.036209 | 0.120 |
| MTC | 5 | 14 | Primary neural | LSTM | 0.008644 | 0.007228 | 0.554 | 0.642 | 0.161 | 100.0 | 0.003659 | 0.002999 | 0.956 |
| MTC | 5 | 14 | Primary neural | GRU | 0.009016 | 0.007371 | 0.563 | 0.667 | 0.164 | 100.0 | 0.004427 | 0.003140 | 0.952 |
| MTC | 5 | 14 | Primary neural | BiLSTM | 0.007806 | 0.006392 | 0.489 | 0.579 | 0.142 | 100.0 | 0.003375 | 0.002314 | 0.964 |
| MTC | 5 | 14 | Primary neural | Causal CNN | 0.017186 | 0.013083 | 0.985 | 1.246 | 0.293 | 100.0 | 0.008090 | 0.002546 | 0.826 |
| MTC | 5 | 14 | Primary neural | CNN-LSTM | 0.007761 | 0.006301 | 0.481 | 0.573 | 0.140 | 100.0 | 0.000375 | -0.000201 | 0.964 |
| MTC | 5 | 14 | Primary neural | CNN-GRU | 0.008956 | 0.007329 | 0.556 | 0.652 | 0.163 | 100.0 | -0.006139 | -0.006711 | 0.953 |
| MTC | 5 | 14 | Primary neural | CNN-BiLSTM compact attention | 0.009188 | 0.007365 | 0.557 | 0.668 | 0.164 | 100.0 | -0.001510 | -0.000656 | 0.950 |

Note. Each target–horizon cell contains 19 primary specifications: 6 statistical models, 6 classical machine-learning specifications (3 models × 2 representations), and 7 primary neural architectures. Neural rows use ensemble-mean predictions across 10 fixed seeds. ASPH indicates age-standardized prevalence of hypertension; MTC, mean total cholesterol; RMSE, root mean squared error; MAE, mean absolute error; MASE, mean absolute scaled error; RMSSE, root mean squared scaled error; sMAPE, symmetric mean absolute percentage error. Direction is directional accuracy. R² is diagnostic only and was not used for ranking. NA denotes undefined.

## Prespecified Neural Ablation Comparisons

Multimedia Appendix Table 2. Prespecified neural attention and capacity ablation comparisons and inference

| **Target** | **h** | **Comparison** | **RMSE difference** | **Bootstrap 95% CI** | **DM-HLN** | **Raw P value** | **Holm-adjusted P value** | **Validity** | **Interpretation** |
| --- | --- | --- | --- | --- | --- | --- | --- | --- | --- |
| ASPH | 1 | Attention | -0.001968 | -0.002162 to -0.001791 | -14.5482 | <.001 | <.001 | Valid | Detected difference |
| ASPH | 2 | Attention | -0.002277 | -0.003113 to -0.001665 | -13.2402 | <.001 | <.001 | Valid | Detected difference |
| ASPH | 5 | Attention | -0.001073 | Not computed | Not computed | Not computed | Not computed | Disabled a priori; descriptive only | Descriptive; no P value |
| MTC | 1 | Attention | 0.000003 | -0.000193 to 0.000198 | 0.0265 | .98 | .98 | Valid | No detected difference; limited power |
| MTC | 2 | Attention | -0.000098 | -0.000251 to -0.000021 | -2.0592 | .06 | .17 | Valid | No detected difference; limited power |
| MTC | 5 | Attention | -0.000548 | -0.001024 to 0.000034 | -0.8900 | .39 | .78 | Valid | No detected difference; limited power |
| ASPH | 1 | Capacity | -0.003959 | -0.004319 to -0.003539 | -10.4831 | <.001 | <.001 | Valid | Detected difference |
| ASPH | 2 | Capacity | 0.006020 | 0.005386 to 0.006929 | 13.8243 | <.001 | <.001 | Valid | Detected difference |
| ASPH | 5 | Capacity | 0.000736 | Not computed | Not computed | Not computed | Not computed | Disabled a priori; descriptive only | Descriptive; no P value |
| MTC | 1 | Capacity | 0.000115 | -0.000207 to 0.000340 | 0.7803 | .45 | >.99 | Valid | No detected difference; limited power |
| MTC | 2 | Capacity | 0.000162 | -0.001227 to 0.001680 | 0.1775 | .86 | >.99 | Valid | No detected difference; limited power |
| MTC | 5 | Capacity | 0.000182 | -0.001312 to 0.002135 | 0.1982 | .85 | >.99 | Valid | No detected difference; limited power |

Note. RMSE difference is comparison model minus the compact-attention reference; negative values favor no attention or Original23K. Reported bootstrap 95% CIs for the ten inferentially valid cells used 5,000 paired repetitions. DM-HLN is the Harvey–Leybourne–Newbold-modified Diebold–Mariano statistic. Holm adjustment was applied separately within each prespecified ablation family. ASPH h=5 (n=5) was descriptive only; its bootstrap CI, DM-HLN statistic, and P values are designated “Not computed” because formal inference was disabled a priori. Non-detection is not evidence of equivalence. Exact unrounded P values are retained in the frozen results.

## Computational Cost and Reproducibility

Multimedia Appendix Table 3. Computational cost, fit counts, and execution reconciliation by model family

| Family | Planned fits | Successful fits | Attempted fits | Failed attempts | Unresolved failures | Cumulative elapsed, s | Estimator fit, s | Median fit, s | Maximum fit, s |
| --- | --- | --- | --- | --- | --- | --- | --- | --- | --- |
| Classical ML | 426 | 426 | 426 | 0 | 0 | 179.534 | 37.878 | 0.052 | 4.805 |
| Neural ablation | 1420 | 1420 | 1420 | 0 | 0 | 1250.407 | 1205.645 | 0.884 | 5.136 |
| Primary neural | 4970 | 4970 | 4971 | 1 | 0 | 5693.467 | 5550.406 | 1.081 | 6.870 |
| Statistical | 426 | 426 | 426 | 0 | 0 | 321.663 | 3.570 | 0.013 | 5.704 |

Note. All 7,242 planned output-producing fits completed. There were 7,243 attempted executions because one primary-neural fit initially encountered a MemoryError during parallel startup; the scientifically unchanged fit was rerun successfully after limiting BLAS/OpenMP threading, leaving zero unresolved failures. Overall experiment wall-clock time was 1903.845 seconds. Cumulative per-fit elapsed time was 7445.070 seconds and cumulative estimator-fitting time was 6797.499 seconds. Cumulative values include parallel work and are not alternative wall-clock durations. The CPU-only system used an Intel Core Ultra 9 185H processor, 16 physical and 22 logical cores, approximately 33.7 GB RAM, and 18 neural workers with one computational thread each.

## Abbreviations

- ARIMA: autoregressive integrated moving average
- ASPH: age-standardized prevalence of hypertension
- BiLSTM: bidirectional long short-term memory
- CNN: convolutional neural network
- DM: Diebold–Mariano
- ETS: error-trend-seasonal state space
- GRU: gated recurrent unit
- HLN: Harvey–Leybourne–Newbold
- LSTM: long short-term memory
- MAE: mean absolute error
- MASE: mean absolute scaled error
- ML: machine learning
- MTC: mean total cholesterol
- RMSE: root mean squared error
- RMSSE: root mean squared scaled error
- sMAPE: symmetric mean absolute percentage error
- SVR: support vector regression
- XGBoost: extreme gradient boosting
